# River basin governance enabling pathways for sustainable management: A comparative study between Australia, Brazil, China and France

**DOI:** 10.1007/s13280-021-01699-4

**Published:** 2022-03-22

**Authors:** Frederick Willem Bouckaert, Yongping Wei, James Pittock, Vitor Vasconcelos, Ray Ison

**Affiliations:** 1 School of Earth and Environmental Sciences,, St. Lucia Campus, University of Queensland, Brisbane, QLD 4072 Australia; 2 Fenner School of Environment and Society, Australian National University, 48 Linnaeus Way, Acton, Canberra, ACT 2600 Australia; 3 Universidade Federal do ABC, São Bernardo do Campo, Santo André, SP Brazil; 4 Applied Systems Thinking in Practice (ASTiP) Program, School of Engineering & Innovation, Faculty of Science, Technology, Engineering and Mathematics (STEM), The Open University, Walton Hall, Milton Keynes, MK7 6AA UK; 5 123 Barclay Street, Deagon, QLD 4017 Australia

**Keywords:** Actor engagement, Diagnostic framework, River basin governance, River basin management, Socio-ecological systems

## Abstract

Successful river basin governance is challenged by actor engagement in the various stages of planning and management. A governance approach for determining priorities for actors for sustainable management was developed, based on a river basin diagnostic framework consisting of four social-institutional and four biophysical indicators. It was applied in river basins in Australia, Brazil, China and France. Actors diagnosed current and target capacity for these indicators, and estimated synergistic influences of interacting indicators. The results reveal different priorities and transformative pathways to achieve basin plan outcomes, specific to each basin and actor groups. Priorities include biodiversity for the Murray-Darling, local water management needs for the São Francisco and Yellow rivers, and improved decision-making for the Adour-Garonne. This novel approach challenges entrenched views about key issues and actor engagement roles in co-implementation of the basin plan under existing prevailing governance models, with implications for engagement and international collaboration on basin governance.

**Supplementary Information:**

The online version contains supplementary material available at 10.1007/s13280-021-01699-4.

## Introduction

River basin governance is increasingly challenged around the world for management of droughts and floods, fair and equitable allocations, wastewater management, and apportioning environmental flows to maintain valuable ecosystem services (Garrick et al. 2014; Tilleard and Ford 2016). Climate change has compounded the problem by magnifying extreme events and introducing non-stationarity (Milly et al. 2008). Over allocation of water in many basins around the world has seen an alarming degradation of biophysical conditions, resulting in a focus on biophysical river restoration outcomes (Turak et al. 2016) and ecosystem services (Costanza et al. 2014; Costanza 2015). Paradigm shifts in river basin management have changed from a hydrocratic top-down command and control model to a decentralised participatory model (Huitema and Meyerinck 2014), recognising that collaborative governance of river basins is paramount (Ansell and Gash 2007). However, to date, actor engagement is often still conducted on case study analysis of specific contexts, but these findings are often not comparable; hence, the knowledge base is too fragmented to support more general insights at the global scale.

Current actor engagement methods such as multicriteria decision analysis (Herath and Prato 2006; Marttunen and Hämäläinen 2008), analytical hierarchy process (Gallego-Ayala and Juizo 2012) and normative scenario approach (Bizikova et al. 2015; Brown et al. 2016) are limited by being highly technical, time consuming, set in a context of economic and utilitarian imperatives (Damiens et al. 2017; Berry et al. 2018) and relatively inaccessible for a wide and diverging actor audience. Strategic and accessible actor engagement in managing basins in a sustainable and equitable way (Daniell et al. 2010; Bos and Brown 2014; Barbosa et al. 2017) is hence limited, and a systemic approach to integrating social perspectives in water governance, is currently lacking, although some tools exist such as the Delphi method (Taylor and Ryder 2003) commonly applied in Brazil (Marrionetti and Santos Canada 2017; Oliveira Mota 2018). A diagnostic framework that can integrate governance and management performance and provide general and comparable insights across a diversity of river basins will hence contribute to global challenges of water security, biodiversity and impacts from climate change.

This work builds on previously published research aimed at linking social institutional capacity and basin biophysical capacity in a diagnostic framework consisting of eight key indicators (Bouckaert et al. 2018). Indicators are useful tools to diagnose the current condition of a river system, providing summary information for managers to direct their attention to high priorities. Setting targets and mapping a trajectory and timeline for achieving them can follow. Moreover, to integrate indicator-based diagnostic capability of actors provides an opportunity for designing an enabling pathway for actor participation in co-implementation. This ensures multiple priorities emerging from the diagnostics can be considered in an integrated approach. In Brazil, the effectiveness of socio-institutional indicators was analysed (Bouckaert et al. 2020), whereas the interaction between socio-institutional and biophysical indicators was investigated in the Murray-Darling Basin (Bouckaert et al. 2021). Thus, this diagnostic framework could be used as a tool to enable diagnostic actor comparisons across basins with diverging social, ecological, and climate characteristics, and reveal how basin governance learnings from one river can be applied elsewhere to allow for greater collaboration, learning and adaptation at a time when global resource management is at crisis point (Neto et al. 2018).

Therefore, the aim of this study is to develop an approach for determining priorities and enabling pathways for sustainable river basin governance and management with an indicator-based actor diagnostic framework at a global context, specifically, the Murray-Darling Basin in Australia, the São Francisco River Basin in Brazil, the Yellow River Basin in China, and the Adour-Garonne Basin in France. It will answer the following research questions:

1. How do the diagnostic indicator priorities differ between actor groups and river basins? What strategies can be identified from these diagnostic priorities?
2. How can actor priorities contribute to an enabling pathway within the constraints of the current governance model for each river basin?
3. How do enabling pathways differ between basins? How do they differ from identified key challenges within each of the basins?

The rest of this paper explains the diagnostic framework, case river basins, data collection and analysis, identified key challenges and indicator profiles, and emerging enabling pathways. The significance for enhanced actor participation and co-implementation is discussed and compared across governance settings associated with basin plans for each of the case basins. International significance and limitations of the method are considered, followed by conclusions.

## Theoretical framework

**Fig. 1:**
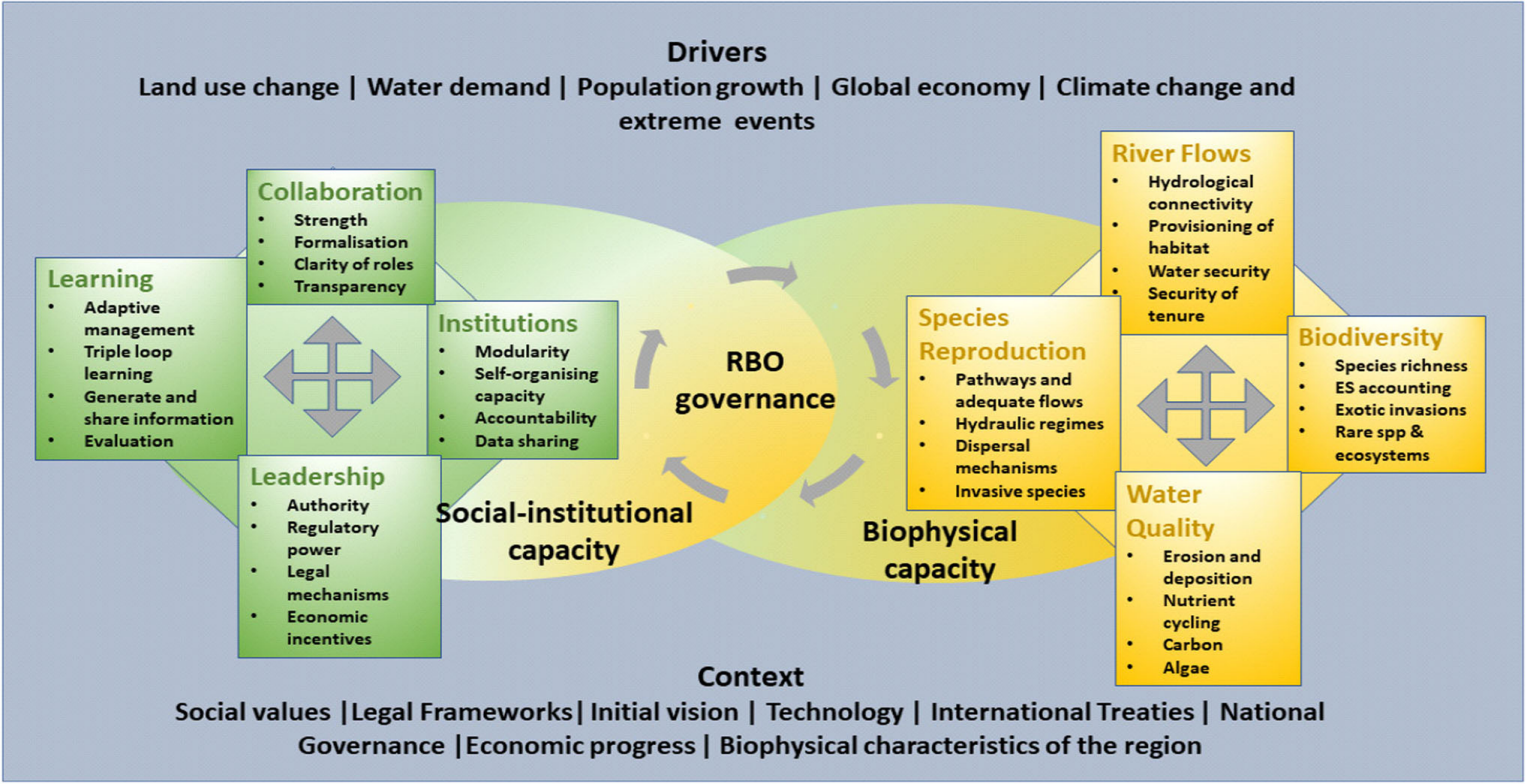
Conceptual framework to assess the capacity of river basin governance and management (Bouckaert et al. 2018)

The water governance approach needs to address the dual challenge of integrating social perspectives in natural resource management and dealing with uncertainties in complex and co-evolutionary socio-ecological systems (Rammel et al. 2007; Duit and Galaz 2008; Akamani 2016). The diagnostic framework developed by Bouckaert et al. (2018) was adopted in this study (Fig. 1). In this framework, the RBO (river basin organisation) is positioned at the interface of two domains, because its function is to implement governance and respond to biophysical feedback mechanisms using adaptive management. The system is open ended, and subject to external drivers and context, over which it has limited control.

Socio-ecological systems are driven by four key dynamic functions that are emerging system properties in continuous interaction. *Connection* is essential to transfer energy, materials, information and creates scalar dependencies and balance. *Structure* represents key components that are essential building blocks with inherent properties, functions and characteristics. *Renewal* ensures the continuity of the system, through reproduction and adaptation. Finally, *Direction* provides the trajectory by which the system adapts and modifies itself towards new ‘end’ points or targets, to maintain its key system functionality, without reaching a tipping point to an alternate state.

The four functions are expressed by key indicators in the social domain (*Collaboration, Institutions, Learning* and *Leadership* respectively), and in the biophysical domain (*River Flows*, *Biodiversity*, *Species Reproduction* and *Water Quality* respectively). Note that *Water Quality* encompasses sedimentary processes shaping channel meandering changes of the river network and habitat niches providing *Direction*. Socio-institutional indicators are regarded as essential for good governance (Ansell and Gash 2007; Bos and Brown 2014), while biophysical indicators relate to biophysical condition influenced by river management and restoration plans (Davies et al. 2012; Lindenmeyer et al. 2012).

The interactions between these indicators can be defined as the relative synergistic or antagonistic influence each indicator has on each of the others while progressing from current towards target condition (after Weitz et al. 2017). Interactions among biophysical indicators mean that changes in ecosystem components are interdependent. For example, *River Flows* will influence *Species Reproduction* by enhancing or diminishing favourable conditions, and over larger time scales affect *Biodiversity*. In addition, *River Flows* will propagate material cycling and hence influence *Water Quality*. But the reverse is also true; at the local scale, an increase in *Biodiversity* can influence *River Flows* and *Water Quality* with cumulative effects on larger spatio-temporal scales. *Species Reproduction* can also influence other biophysical indicators, by contributing or reducing (in the case of exotic species) *Biodiversity* or impacting *Water Quality* such as through blue green algal blooms. This is outlined in the hierarchy theory (McLoughlin and Thoms 2015), and can be used for synergistic management strategies. Similarly, governance indicators are interdependent. Strong *Leadership* will promote direction and can (dis)advantage *Collaboration*, depending on the circumstances. Efficient but nimble *Institutions* will assist in both long-term and short-term management, whereas *Learning* will foster data collection, information sharing and *Collaboration*. Governance indicators also influence biophysical indicators through policy settings, but do not fully control the biophysical world. Changes in biophysical conditions such as droughts, floods, storms can cause disaster vulnerability requiring short-term or long-term resilience strategic responses (Jackson et al. 2017). Long-term changes are often more difficult to diagnose, as they may not become apparent for decades, for example gradual loss in *Biodiversity.*

The diagnostic ability of this conceptual framework lies in scoring indicators for current and target condition, and rating synergies in progress towards target. Aggregated actor scores can be used to design enabling pathways for engagement to achieve target condition objectives.

## Materials and methods

### Case river basins

**Fig. 2:**
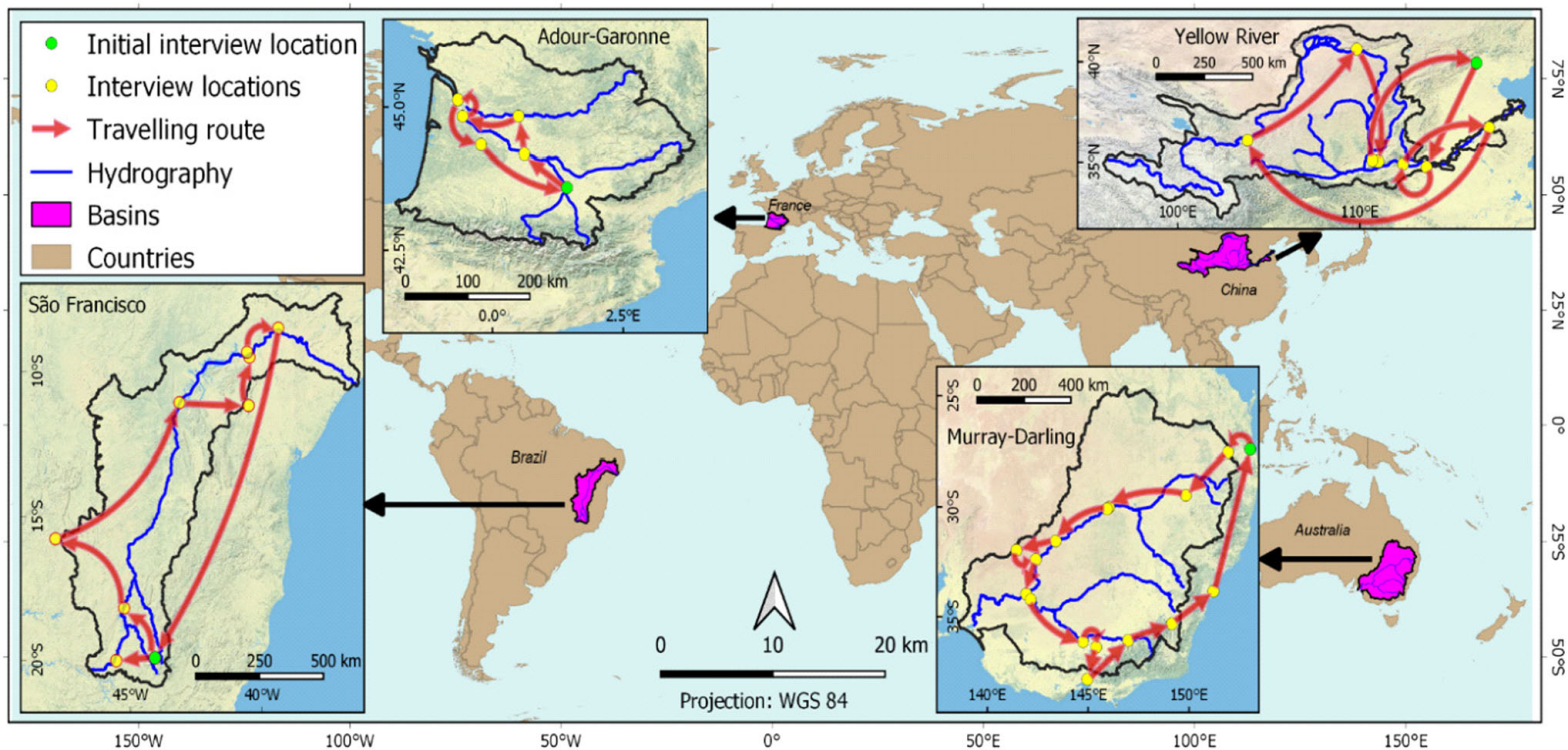
Location and travel trajectory of the four river basin case studies

Four case river basins were selected based on the following principles: (a) the majority of river basins are embedded within the governance provenance of nation states, (b) each river basin represents a significant economic resource for the country, has a RBO or basin committee, and faces unsustainable resource management issues resulting in declining river health conditions, (c) governance settings range from elected representation of actors to centralised hierarchical institutions across four continents, (d) basins cross intra-federative borders, such as states or provinces, and (e) each river basin is part of significant water resource management reform. Additional considerations (travel, assistance with logistics and language barriers) resulted in the selection of the Murray-Darling Basin in Australia, the São Francisco River Basin in Brazil, the Yellow River Basin in China, and the Adour-Garonne Basin in France (Fig. 2).

The Murray-Darling Basin is one of the most significant river basins in Australia, covering an area greater than 1 000 000 km^2^ with 30 000 wetlands, 16 of which are internationally significant through Ramsar listing and providing an annual agricultural industry worth $2.4b and drinking water to over 3 m people (MDBA 2020). The southern basin is heavily regulated through major storage reservoirs and water is over allocated to irrigation (Wei et al. 2011). The Basin Plan (2012) legislated under the Water Act (2007) and implemented by the MDBA (Murray-Darling Basin Authority) aims to return 2,750GL of water to the environment through accredited water resource plans implemented by the state governments. Implementation has been controversial and politicised, under influence of powerful actors (Wentworth Group of Concerned Scientists 2018). In the northern basin, stewarding environmental flows in an unregulated system, where private property floodplain harvesting is inadequately regulated, has proved particularly challenging under drought conditions (Australian Academy of Science 2019; Vertessy et al. 2019).

Institutionalised engagement with civil society is poorly structured, biased towards the private sector and does not allow for equitable actor representation (Grafton and Williams 2019). Tensions between state and federal governments and MDBA’s conflicting roles of regulation, implementation, and service provision instigated a call for additional institutional reform (Productivity Commission 2018). The new challenges arising during the Basin Plan implementation provide a useful case study.

The São Francisco River Basin in Brazil has economic significance for agricultural irrigation (77% of water consumption), industry, mining, and hydropower, especially in the relatively wealthier upper basin state of Minas Gerais (Lee et al. 2014). The river has an average flow of 2,880 m^3^ and serves 19 million basin inhabitants. The main river is under the jurisdiction of the federal government, with subsidiary delegation of certain regulatory functions to a basin agency that follows the directions given by the CBHSF (São Francisco River Basin Committee). The CBHSF consists of 62 elected representative members from civil, public, and private sectors of society from each of the states, resulting in a highly complex governance structure. While the upper basin has abundant water resources, the middle, and lower states are much poorer with very few tributaries and a semi-arid savannah ecology.

Three major dams (Três Marias, Sobradinho, and Itaparica) provide large reservoirs used for irrigation, hydropower, and a controversial water diversion project to the northern states of Ceará, Paraíba, Pernambuco and Rio Grande do Norte, which caused a governance crisis between the CBHSF and the federal government (Empinotti et al. 2018). Despite opposition from the CBHSF, the ANA (National Water Agency) approved the water diversion project in 2007 (Roman 2017). Since then, the CBHSF has reconciled its position with ANA, but the basin management plan (CBHSF 2016) and the restoration program (MMA 2004) are still developed in parallel rather than being integrated. The decision-making power of the basin committees in Brazil is still conditional on obtaining federal funding thereby weakening the representative governance model and creating significant tension and distrust. The governance challenges between upper and lower basin states and between the federal government and the basin committee make it a suitable case study to test our proposed approach.

The Yellow River is the second largest river in China (> 5400 km long), and often called the cradle of China’s civilisation, but also China’s sorrow, due to its history of massive floods that caused a lot of death and suffering. Its catchment contains around 9% of the population and 17% of the agricultural area of the whole nation (Giordano et al. 2004). The upper reach includes the river origin to Lanzhou, which contributes 56% of runoff (Song 2011). The middle reach runs through the Loess plateau, where a massive revegetation project has restored the riverbanks and significantly reduced the sediment loads, responsible for the river’s name. The lower reach is characterized by its unique suspended riverbed relative to the floodplain, which is the most populated area of the basin. Water allocation is under the authority of the Yellow River Conservancy Commission (YRCC) and remains challenging. In 1997 the river ceased flowing for over 700 km in 226 days, in part resulting from the revegetation project in the Loess plateau (Chen et al. 2020).

Water governance is complex, being characterised by limited capacity for enforcement by the YRCC, and poor integration with local water affairs bureaus at county level responsible for water supply, urban water savings, flood management and wastewater treatment (Shen and Speed 2009). Economic priorities often prevail in a competition between district counties to attract investment. In an effort to control this problem, River Chiefs are being appointed for every river for better protection of water resources (Ministry of Water Resources 2018). Besides water quality issues, irrigation demand under drying conditions, sediment management and flood control, the additional drivers of urbanisation and rapid industrialisation (Webber et al. 2008) increasingly challenging basin governance, making it an interesting case study to examine actor perspectives.

The Adour-Garonne Basin is one of seven river basins in mainland France, situated in the southwest of the country. The basin consists of a network of 120 000 km long streams and spanning an area of 117 650 km^2^, 400 km of coastline and three major estuaries and is home to 7.5 million people. Twenty percent of its territory is urban, passing through Toulouse in the region of Occitanie, midstream, and Bordeaux, in the region of Nouvelle Aquitaine, near Gironde, the estuary (République Française 2019). Agriculture uses 80% of all consumption, and the basin has the largest structural water deficit in France (Mazegga et al. 2014). The Comité Adour-Garonne (CAG), established under the French Water Law 1964 has 135 members and consists of 40% of elected local government officials, 40% of water users and 20% of State representatives (Colon et al. 2018). It is responsible for management and implementation of intervention programmes, and the basin-wide Master Plan for Water Development and Management (SDAGE) and sub-basin Plans for Water Development and Management (SAGE) for each of the major tributaries (Yang et al. 2013), which emerged under the new 1992 Water Act for sustainable development (Agence de l’Eau Adour-Garonne 2020).

In 2006, the new norms from the European Water Framework Directive were incorporated in the revised Water Act; the emphasis is on ecosystem-based objectives, water quality targets and results oriented management. Conflicting agendas of different actor groups have often led to favouritism and have caused impasse in decision-making (Colon et al. 2018). Ongoing administrative reforms mean that the regions will get a greater say in streamlining the diverse SAGE catchment plans constituted by relevant council representatives. In addition, the quantified flow and ecological targets and deadlines under the EU Water Framework Directive allow each EU country to use its own programs for implementation, but appear difficult to comply with, not only within the Adour-Garonne Basin but also across many basins in European countries (Fernandez et al. 2014). This presents an interesting challenge for our fourth case study.

### Data collection

Actors were interviewed in the upper, middle and lower parts of the basins, and location visits contributed also to a geographic understanding of the basin characteristics (Fig. 2). Actor contacts were sourced from RBOs, internet searches and the snowballing method (Cohen and Arieli 2011). Participants self-identified with one of ten actor categories: decision-makers, irrigators, scientists, members of an NGO, RBO subsidiary (members of government responsible for water management not part of the RBO), hydropower/water supply, ecosystem beneficiaries (such as traditional fisherman and riverine small-farming communities), RBO staff, and members living in the basin (community groups). Only five categories had consistently more than one participant across four basins and were included in this study together with the mean of all five group scores (Table 1). Obtaining informed consent was complemented by assuring anonymity of the results.

**Table 1:** Participants interviewed in each basin, by actor group

Actor groups	Murray-Darling Basin, Australia	São Francisco Basin, Brazil	Yellow River Basin, China	Adour-Garonne Basin, France
Decision-makers	3	6	3	4
Irrigators	3	4	2	2
Scientists	8	5	4	2
NGO	5	4	2	4
RBO subsidiary	4	6	5	5
Sum of all included	23	25	16	17

After explaining the framework and purpose of the research, qualitative and quantitative data were obtained using semi-structured interviews with a standardized list of detailed questions (Table S1) except for the basin specific vision (Table S2) and question on major policy initiatives (Table S3). The qualitative data consisted of open-ended questions related to various aspects of how the RBO could implement the basin plan and perceived key challenges. These questions provided context and complementarity to the diagnostic assessment of the basin. For the eight key indicators, participants were asked to score on a five-point scale (Likert 1932) current and target capacity from *Very Poor* to *Very Good*, based on a descriptive rubric (Table S4). Target date for this assessment was aligned with year of completion for the relevant basin plan or policy (usually a period between 5 and 10 years). Diagnostic scores were aggregated by actor group. The difference in score between target and current (T–C) values was used to indicate distance (difficulty) to reach target (Fig. S1). Next, participants rated the synergistic or antagonistic influence of indicator progress towards target on other indicators (after Weitz et al. 2017) using a scale of *Counteracting* (− 2), *Constraining* (− 1), *Consistent* (0), *Enabling* (1) and *Reinforcing* (2). The aggregated scores were used to calculate priorities for constructing enabling pathways (Fig. S2).

### Data analysis

Key challenges for actor groups were derived by summarizing individual qualitative responses in a thematic statement. These interview statements were grouped into thematic categories. For each group in each basin, a number of themes emerged that were identified as key challenges, which were then attributed to corresponding diagnostic indicators.

Diagnostic and influencing scores were averaged by actor group to allow comparisons between basins. The results of averaged actor scores assume to express a collective view on basin diagnostics. This collective view provides a window on actor expectations vis-a-vis their own and other groups’ priorities and engagement. High (T–C) scores signify a large difference between target and current condition. This can result from high target expectations and moderate current conditions, or poor current conditions and moderate to high target expectations. Regardless, a larger value represents a larger distance between both, which makes it more challenging to achieve the target condition by the completion target date. Where (T–C) ≤ 0, progress towards target is either not possible or already achieved ((T–C) = 0) or will inevitably decline, despite best efforts, though perhaps at a slower rate than would have happened without management interventions ((T–C) < 0).

Enabling pathways to complete indicator targets were developed using the following criteria in order of priority:

1. Selecting the hardest target challenges, indicated by the three largest (T–C) difference values for indicators. The largest value is the starting priority for an actor group to focus on, descending towards the next hardest target indicator and the next. Three indicators provide a two-step pathway, although they are not always strongly connected by an influencing link in which case the next strongest priority may be discontinuous with the previous one (Data analysis Step 1, Fig. S2), or branch in two directions (two equal second largest values).
2. The indicator exerting the strongest interactive positive influence (enabling and reinforcing) according to one or more actor groups is identified from the influence matrix using largest values. This indicator has a strategic advantage when designing the enabling pathway (Data analysis Step 2, Fig. S2).
3. Cumulative strongest influences (largest values) of one indicator across all others by actor group were also determined (Data analysis Step 3, Fig. S2).
4. The combination of Criteria 1, 2 and 3 was used to design primary and secondary influence links between indicators, denoted by thick and thin arrows respectively for each actor group, and combined in one enabling pathway schema for each basin. Indicator node boxes were coded by shape and colour to denote their influence by actor group (Fig. S2).

## Results

### Open-ended questions on key challenges for actor groups in each river basin

The views of actor groups are closely linked to the basin plan objectives of each river basin, and therefore differ between basins despite an assumption that particular groups may pursue similar interests universally. They also differ from priorities emerging from diagnostic condition scoring. Actor views reflect the diversity of engagement of these groups and the way they identify key challenges, which are context specific. When comparing how challenges are assigned to indicators, the array of challenges for an indicator is wide-ranging across basins and actor groups (Table 2). Hence, aligning challenges with indicators results in different emphasis by actor groups across basins. For example, in the Murray-Darling Basin, three groups emphasise mainly governance indicators as key challenges: decision-makers, NGOs and scientists. From those same groups in the São Francisco Basin, only scientists and NGOs identify *Collaboration* as a challenge; the rest are biophysical indicators, whereas in the Yellow River Basin only NGOs identify *Collaboration* and only decision-makers do in the Adour-Garonne.

**Table 2:** Key challenges identified by actors in the four river basins

Actors	Identified challenges	Corresponding indicators
	*Murray-Darling Basin*	
Decision-makers	Compliance with the Basin Plan is critical and will require further institutional reform. Reform should also include broader catchment management objectives to complement the objectives of the Basin Plan	Institutions
Irrigators	Further implementation and tracking of results for environmental flows are the biggest challenges	River Flows
NGOs	Better integration at all levels is required under a strong MDBA leadership; this is conditional on MDBA becoming truly independent	Leadership Collaboration
RBO subsidiaries	Fairer operating rules for water allocation balancing environmental and human water use	River Flows
Scientists	Institutional reform is needed to better integrate local catchment management plans and foster better collaboration and the building of knowledge infrastructure for evidence-based decision-making and enforcement of compliance	Learning Collaboration Institutions
	*São Francisco River Basin*	
Decision-makers	A basin-wide strategy needs to integrate local water needs and water transfer projects, based on rational decisions. In the long term, increased water demands cannot be met; a paradigm shift is needed but the pathway is not clear	River Flows
Irrigators	Water payment needs strengthening by CBHSF and be made transparent in terms of allocation for river basin restoration projects. The latter will require better integration with local councils	River Flows Biodiversity Institutions
NGOs	CBHSF should reach out more to actors, to allow a bottom up and more decentralized approach. Social justice issues and environmental issues need much greater priority based on a diversity of localized needs	Collaboration
RBO subsidiaries	Basin-wide understanding should be improved by quantifying water use and monitoring, and more actor representation is necessary	River Flows Institutions
Scientists	Multiple issues were mentioned: restoration versus irrigation; increase actor parities; social justice and ecological sustainability; institutional structure versus political interference, water allocation & interstate Water Pact, ensuring minimum flow between upstream and downstream states	River Flows Institutions Collaboration
	*Yellow River Basin*	
Decision-makers	More efficient water allocation, enforcement of regulations and delineation of responsibilities are key priorities to manage floods (short-term) and sediment (long-term), and to integrate local versus system-wide planning, including Lower Yellow River ecology challenges	River Flows Water Quality Biodiversity
Irrigators	Flexibility in water allocation and fees could assist in greater collaboration and meeting the YRCC objectives	River Flows Collaboration
NGOs	Pursuing a uniform and fair approach to manage the difference between environmental and human use of water with better protection for wetlands and greater dialogue for management input by NGOs	Collaboration Biodiversity
RBO subsidiaries	Key governance challenges include institutional reform for better integration at national, basin, regional and local level; water allocation and levies; monitoring and regulation	Institutions River Flows
Scientists	Implementing knowledge gained at the basin scale remains challenging, but also knowledge gaps and low awareness of localized needs and problems, such as water pollution	Learning Water Quality
	*Adour-Garonne River Basin*	
Decision-makers	Despite institutional reform to engage with regional authorities, there is a lack of strategic direction and lack of actor ownership of water governance	Collaboration
Irrigators	Water storage should be actively managed for dealing with climate change rather than trying to restore natural flows; we should adapt to changing ecological conditions, whilst maintaining irrigation water use	River Flows
NGOs	Economic interests maintain and increase artificiality; we need to rethink agriculture to move to a much more sustainable paradigm, by using interdisciplinary science to develop predictive scenarios and options. Stronger regulation and connections with local authorities will be required to achieve this	Biodiversity River Flows Leadership
RBO subsidiaries	Special interest groups (agriculture) are preventing the development of a common long-term vision, which aligns with the EU Water Framework Directives; this will also require integrating planning for the big water cycle with those of the small water cycle, over which CAG has no influence at present	Collaboration Institutions
Scientists	There is a need to educate people about investing in conservation and biodiversity protection and to counter lobbying from agriculture	Learning Biodiversity

### Basin indicator priority profiles for actor groups in each river basin

**Fig. 3:**
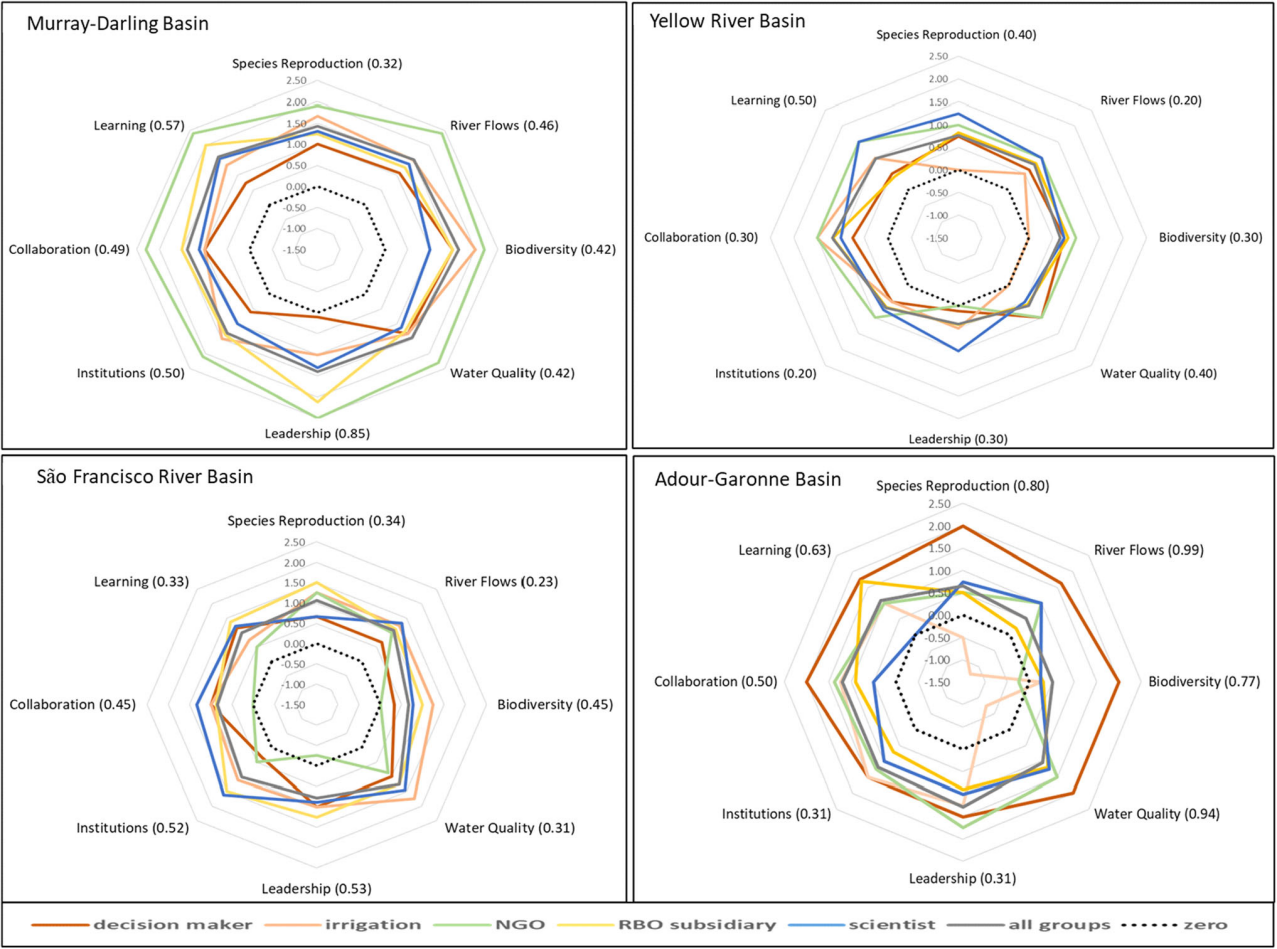
(T–C) score values for actor groups for the four river basins (standard deviation values for each indicator score variation in brackets)

Actor profiles for (T-C) scores are presented in different colours in a spider diagram for each river basin for visual comparison of scores (Fig. 3); the black dotted profile indicates zero values for reference.

The Murray-Darling is the only basin where all scores are greater than zero, meaning that improvement in all indicators is possible, unlike for some of the other basins. In the Murray-Darling diagram (upper left), there are large differences between actor profiles, with NGOs expressing the largest challenges for all indicators indicated by high scores. In contrast, decision-maker scores are some of the lowest, suggesting they have more confidence that challenges can be managed despite *Biodiversity* and *Water Quality* scoring higher than the others. Variation between scores is less prominent for other actor profiles, with the possible exception of higher scores for *Leadership* and *Learning* for RBO subsidiaries. Overall, large variability between actor profiles suggests that communication and shared values are lower compared to more similar profiles in other river basins.

In the Yellow River, irrigators believe that for *Species Reproduction*, *Biodiversity* and *Water Quality* progress cannot improve (diagnostic scores for current and target are identical, see Fig. S1), whereas NGOs indicate no change for *Leadership* (zero difference between current and target score, both = 5). Irrigators and NGOs mark *Collaboration* higher than other groups, suggesting they identify this as a larger challenge. Overall, scores are slightly more similar between actors than those for the São Francisco Basin, with lower scores for biophysical indicators. This is due to current condition scores being moderate, with a modest target ambition of achieving good condition (one increment) for most biophysical indicators (Fig. S1).

In the São Francisco Basin *Leadership* is scored as declining by NGOs (negative score), and *Collaboration* and *Biodiversity* as static. In the case of *Collaboration*, the diagnostic score is high (3.88) and remains the same for current and target, whereas for *Biodiversity* it remains low (2.00) (Fig. S1). Scores are lowest for NGOs compared to others except for *Species Reproduction* and *River Flows* where they align with others, and for *Institutions* where decision-makers score lower. Other profiles look more similar, except for the lower scores from decision-makers for *Institutions*, *River Flows* and *Biodiversit*y, suggesting they are viewed as easier to achieve by this group. This contrasts with scientists’ scores for *Institutions* and *Collaboration*, harder to achieve for this group. Overall scores are lower than for the Murray-Darling Basin, with smaller variation between actors, suggesting a higher degree of agreement. The lower scores suggest that targets are easier to achieve, not because targets were set lower, but because the condition of the basin is assessed as less degraded.

In the Adour-Garonne, the profile from decision-makers stands out, with higher scores for most indicators pointing to high challenges, in particular for biophysical indicators, and *Collaboration*. Irrigators score *Species Reproduction, River Flows* and *Water Quality* as declining despite best management efforts, but in contrast they see a minimal target improvement in *Biodiversity*. Scientists perceive no improvement in *Learning* (condition score 3—Modest, see Fig. S1) and identify *Water Quality* and *River Flows* as the largest challenges. Overall, actor views in this basin have the largest variability in scores (Table 3), which may contribute to the perception of large challenges in *Collaboration* and *Learning*. Less uniformity in scores and larger value ranges, similar to the Murray-Darling Basin, will make water governance more challenging, as ‘accommodating differences’ under a shared vision (Ison et al. 2013) becomes more difficult.

**Table 3:** Standard deviation values for range of actor (T–C) scores

SD across actor groups	Murray-Darling basin	São Francisco Basin	Yellow River Basin	Adour-Garonne Basin
Species reproduction	0.32	0.34	0.42	0.80
River flows	0.46	0.23	0.20	0.99
Biodiversity	0.42	0.45	0.35	0.77
Water quality	0.42	0.31	0.37	0.94
Leadership	0.85	0.53	0.35	0.31
Institutions	0.50	0.52	0.19	0.31
Collaboration	0.45	0.45	0.29	0.50
Learning	0.57	0.33	0.47	0.63
Across all	0.16	0.11	0.10	0.27

Indicator profile scores reveal large variability between actors for some indicators, and closer alignment for others, both within and between river basins. Table 3 summarises this information as standard deviation (SD) values for each indicator and river basin.

Across all indicators, actor scores are much more similar in the Yellow River Basin and the São Francisco River Basin, despite their different governance settings. The Murray-Darling Basin ranks third, as a result of the wide divergence between NGO and decision-maker scores. In the Adour-Garonne, the SD is highest because of negative scores for *River Flows* by irrigators, and *Biodiversity* by NGOs. By indicator, the lowest value and hence greatest agreement is for *Institutions* in the Yellow River Basin (0.19) and the highest for *River Flows,* in the Adour-Garonne (0.99), attributable to the contrast in scores between decision-makers and irrigators in that basin.

### Enabling pathways

Enabling pathways are based on challenges to reach indicator target condition, starting from current condition, as perceived by an actor group. The largest three challenges (T–C values) become the priority focus in descending value, creating an enabling pathway to achieve synergistic outcomes for each indicator.

**Fig. 4:**
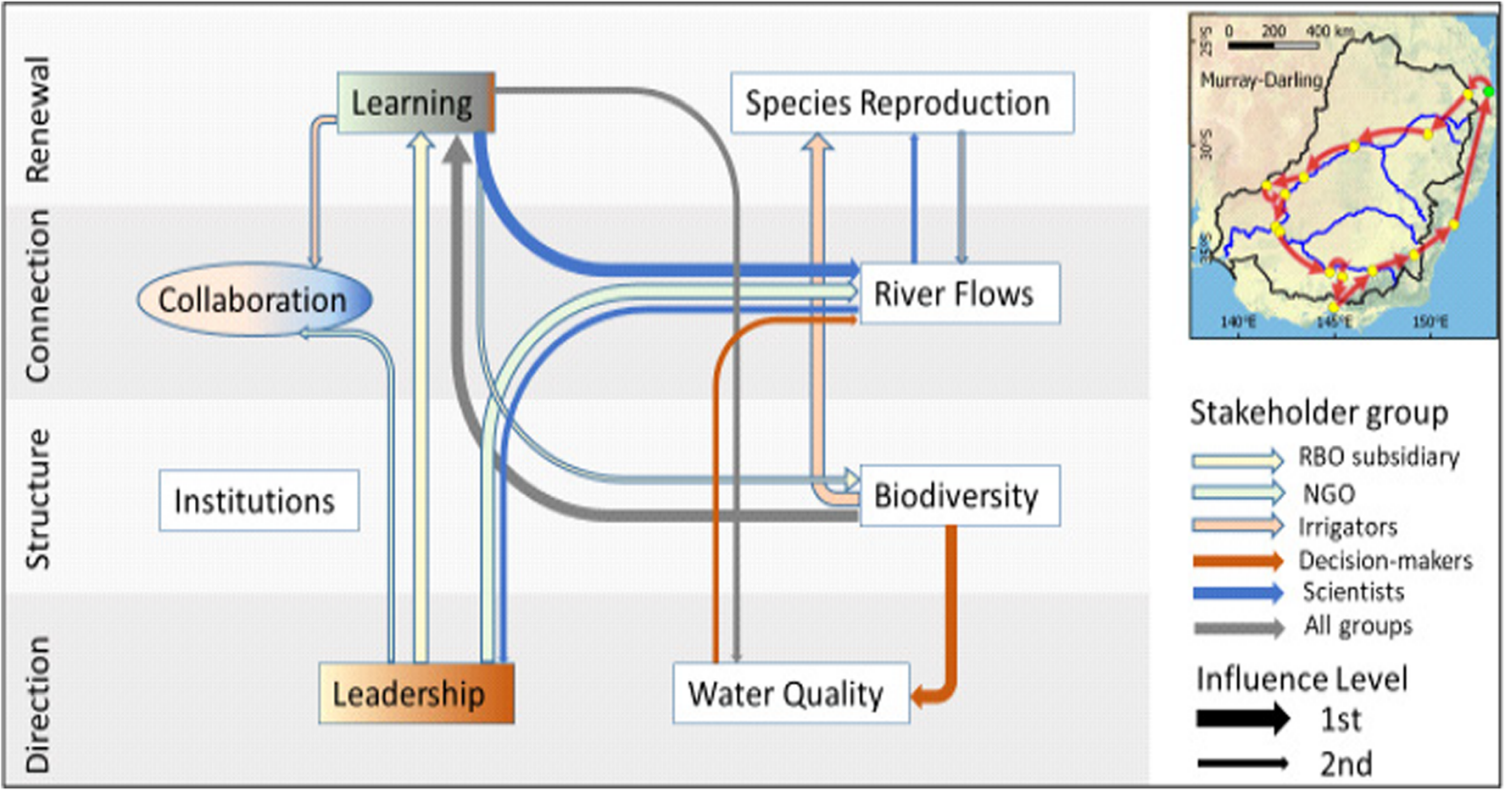
Enabling pathway for the Murray-Darling Basin

In the Murray-Darling Basin (Fig. 4), *Leadership* and *Learning* are two governance indicators that interact to influence *River Flows*, although the latter also influences and is influenced by *Species Reproduction* and *Water Quality*. For scientists, *Learning* leads to improved *River Flows*, which further influences *Leadership* and *Species Reproduction*. NGOs focus on *Leadership* to influence *River Flows* and to enhance *Collaboration*, whereas RBO subsidiaries direct *Leadership* to *Learning* to improve *Biodiversity*. Restoration management of *Biodiversity* for ‘All groups’ will improve *Learning,* which will improve *Water Quality*. Decision-makers prioritise *Biodiversity* to influence *Water Quality,* which then affects *River Flows*, whereas irrigators follow the *Biodiversity Species* to *Reproduction* to *River Flows* pathway, but also use *Learning* for greater *Collaboration.*

**Fig. 5:**
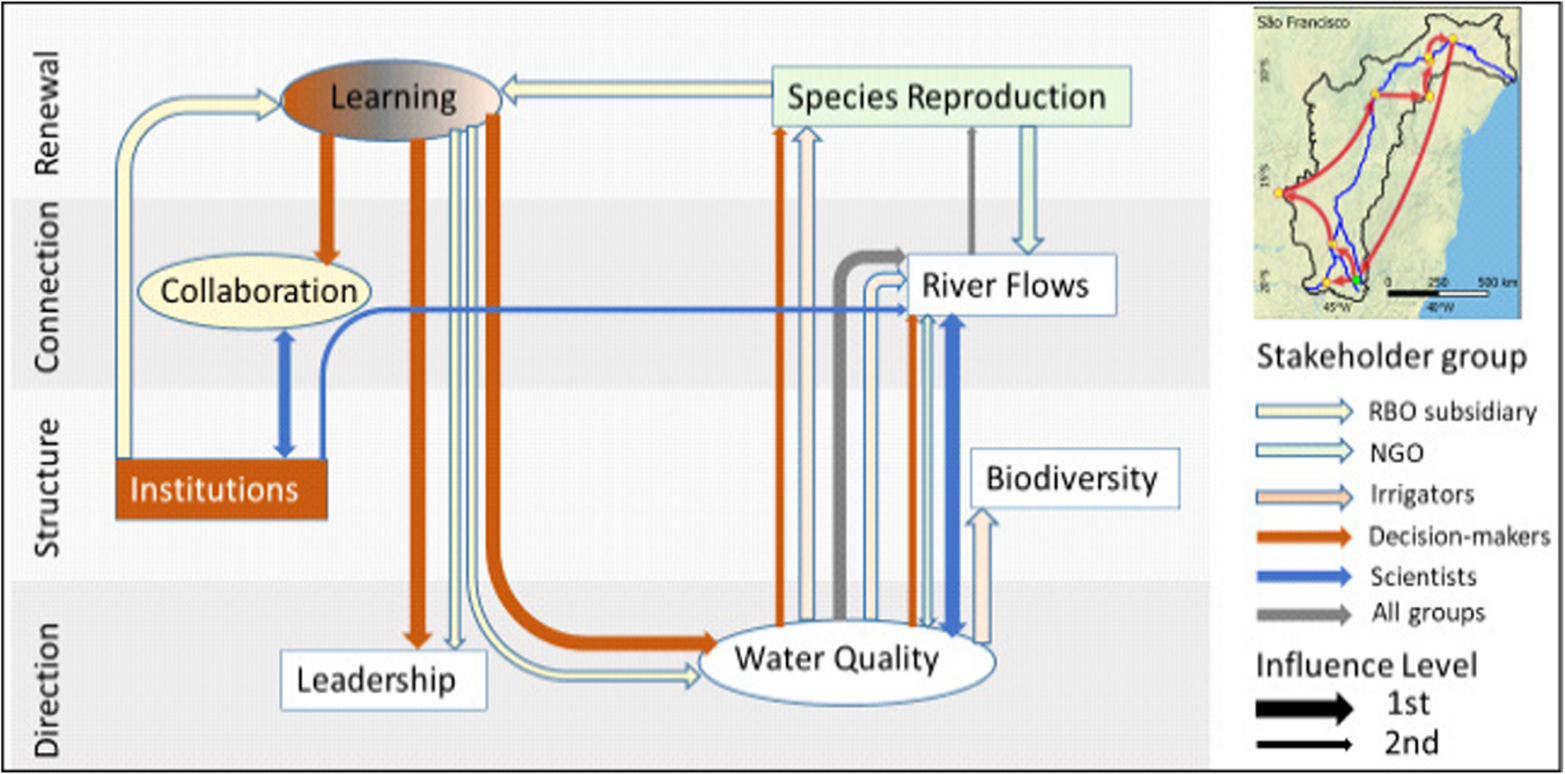
Enabling pathway for the São Francisco River Basin

The pathway for São Francisco (Fig. 5) focusses on interaction between *Water Quality* and *River Flows*, with spin-offs for *Biodiversity* and *Species Reproduction*, and connecting to governance indicators through *Learning*. For NGOs *Species Reproduction* has the strongest influence, for RBO subsidiaries it is *Collaboration* and for decision-makers *Institutions*. For NGOs, the focus is on improving *Species Reproduction*, enhancing *River Flows* and in turn *Water Quality. Water Quality* is the starting priority for ‘All groups’, scientists and irrigators. It also receives influence from *Learning* from decision-makers, with secondary priorities going to *River Flows* and *Species Reproduction*. Only three groups are prioritising governance indicators: decision-makers use *Learning* to improve *Leadership* and *Collaboration*; RBO subsidiaries use *Institutions* and *Species Reproduction* to enhance *Learning* and further influence *Leadership*; scientists are forging a two-way connection between *Collaboration* and *Institutions* to progress *River Flows*, and the interaction with *Water Quality*.

**Fig. 6:**
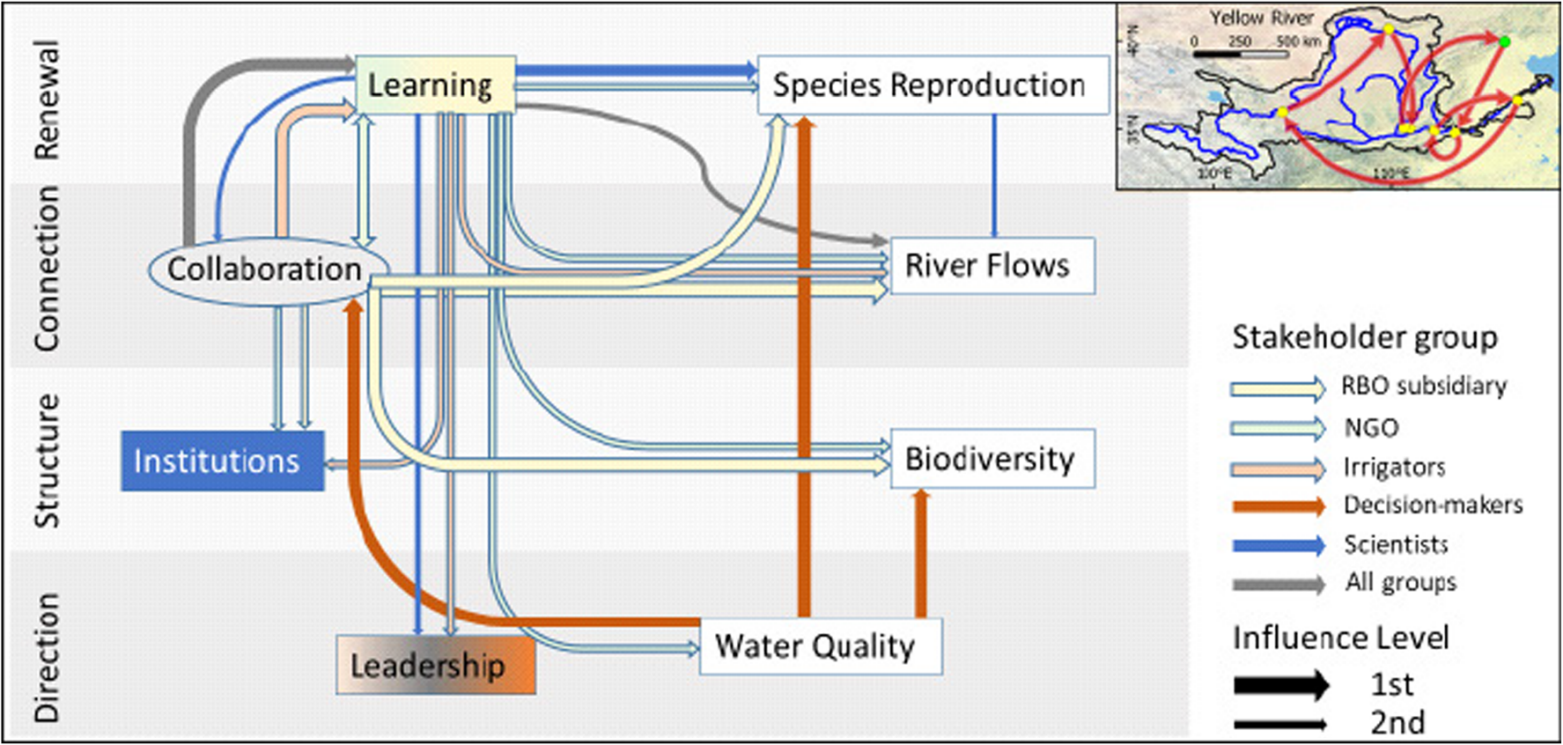
Enabling pathway for the Yellow River Basin

The Yellow River pathway (Fig. 6) centres on *Learning* as a key indicator, being the strongest influence on other indicators according to NGOs and RBO subsidiaries. *Learning* is the starting priority for NGOs and enhances *Collaboration*, and is directed as a secondary priority mainly towards all biophysical indicators by NGOs and to other governance indicators by irrigators and scientists. First priority for scientists directs *Learning* on *Species Reproduction* to inform improved targets for *River Flows*, whereas for ‘All groups’ and irrigators, *Learning* to *River Flows* take second priority. A condition for progressing *Learning* is stronger *Collaboration*, the first priority for ‘All groups’, irrigators and RBO subsidiaries. In contrast, decision-maker priorities start with *Water Quality* to enhance *Biodiversity* and *Species Reproduction*, but also to influence *Collaboration*.

**Fig. 7:**
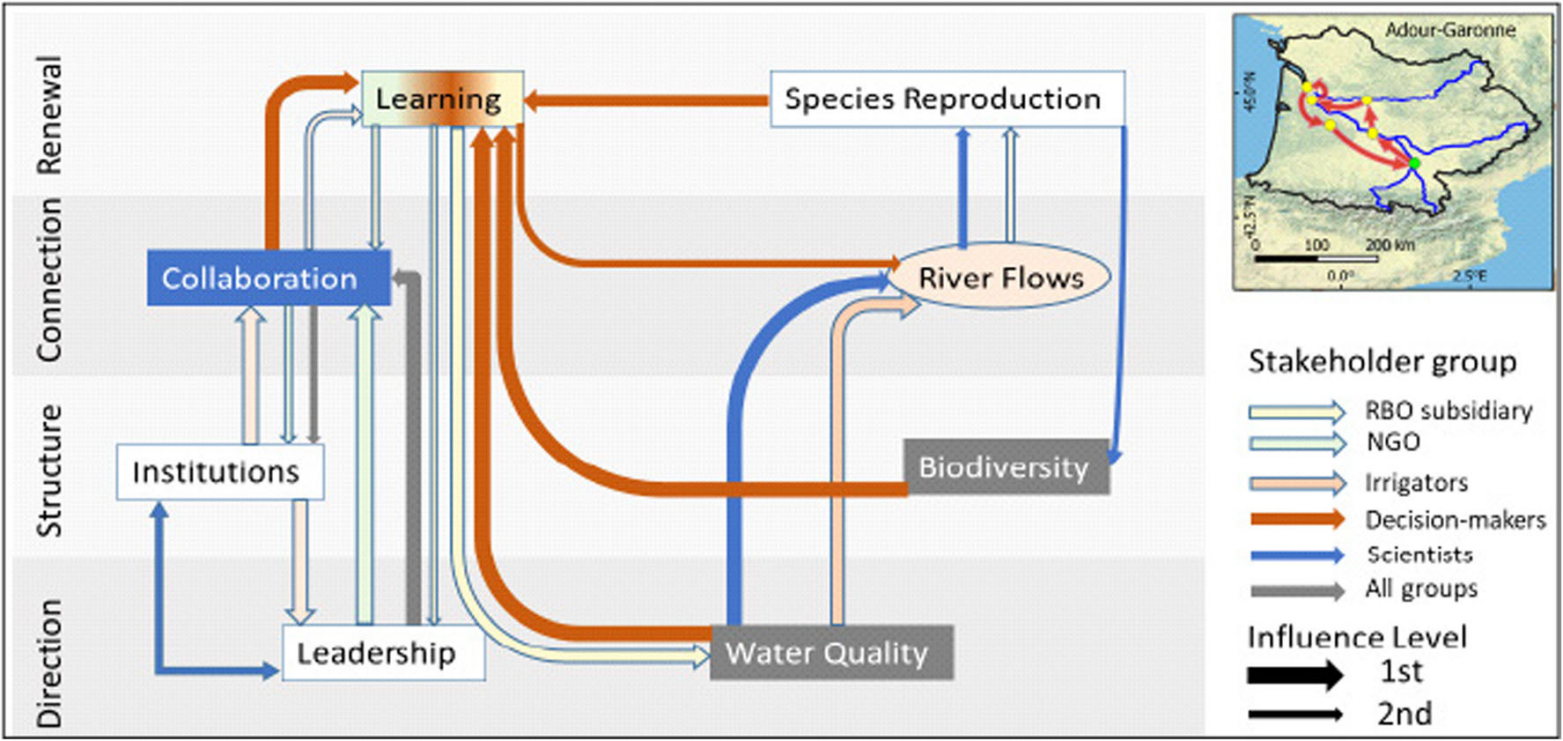
Enabling pathway for the Adour-Garonne Basin

In the Adour-Garonne enabling pathway (Fig. 7) the central indicator is *Collaboration*, which interacts in multiple ways for many groups with other governance indicators *Institutions*, *Leadership* and *Learning*. *Learning* is the critical connection point with biophysical indicators, three of which are of particular interest to decision-makers: *Water Quality*, *Biodiversity* and *Species Reproduction*, which in turn would influence improvement of *River Flows*. The focus on governance indicators is significant for ‘All groups’, which aim to improve *Leadership* to enhance *Collaboration* and consolidate this in enhanced *Institutions*, a pathway that is also significant for NGOs. For decision-makers improving *Collaboration* is the key to *Learning* from *Biodiversity*, *Water Quality* and *Species Reproduction* which may benefit *River Flows*. For ‘All Groups’ and NGOs in particular improvement starts with enhancing *Leadership*. For irrigators, the pathway follows the opposite direction, with *Institutions* improving *Leadership* and *Collaboration*, followed by *Learning.* RBO subsidiaries prioritise *Learning* also towards *Water Quality*, whereas scientists perceive improving *Water Quality* to influence *River Flows*, continuing to *Species Reproduction* and finally to *Biodiversity*.

## Discussion

### Diagnostic indicator priorities, key challenges and governance models [816]

Diagnostic indicator priorities for the Murray-Darling Basin suggest that scores from decision-makers may underestimate the challenges to reach target, while the NGO group may overestimate the challenges ahead (Fig. 3). Key challenges identified by decision-makers require institutional reform to improve compliance issues (Table 2). However, institutional reform to improve compliance may ignore the complex relationship between over-allocation and climate change, overlooked in the Basin Plan (Pittock 2009; Alexandra 2017). The NGO group may base its diagnosis on its desire for better integration at all levels under MDBA coordination (*Leadership* and *Collaboration*, Table 2). This may relate to feeling excluded from transparent decision-making and access to adequate information, explaining difficulty in reaching targets (high scores). The scoring profile of scientists shows a similar but consistently lower pattern across indicators, suggesting more informed views on effort required to reach targets. Hence, information bias can exist across different actor groups, influencing diagnosing problem definition and decision-making. In the Murray-Darling Basin’s centralised governance model, decision-makers have the power to arbitrarily consult with actors and therefore ignore views that may complicate, slow down, or inconvenience the timeline for implementation of the Basin Plan in which government has invested considerable resources and effort to reach social endorsement. As progress with implementation of the Basin Plan may not deliver the intended environmental outcomes, social legitimacy may increasingly be questioned by actors who feel excluded from co-implementation.

In contrast to the Murray-Darling Basin, the NGO group of the São Francisco Basin has the lowest scores of all groups, including two ‘zero’ scores for *Collaboration* and *Biodiversity* and one negative score for *Leadership* (Fig. 3), indicating that no improvement is expected for those indicators and even a potential decline for *Leadership* (the current score being 3.75, moderate to good, Fig. S1). Surprisingly, despite the ‘water parliament’ governance model where NGOs are represented on the RBO committee, this group feels powerless, as the key challenge identified a need to reach out to more actors and allowing a bottom-up approach (Table 2), to give social justice and environmental issues greater prominence (*Collaboration*). Undoubtedly, this is the result of the legacy of the water transfer project, which was decided unilaterally by the federal government (Andrade et al. 2009), against opposing views of the RBO committee at the time. This illustrates that legal actor representation in itself provides no guarantee to avoid actor backlash and disengagement (Empinotti 2007). The Yellow River Basin has the lowest divergence in actor profiles of all river basins, with governance indicators rating as slightly more challenging than biophysical indicators. In particular, *Learning* has the widest range of views, with scientists and NGOs identifying this as a large challenge. In contrast, *Institutions* has the lowest SD value and low scores (Table 3, Fig. S1); this is not considered a priority despite RBO subsidiaries identifying it as a key challenge. Interestingly, low confidence of irrigators in improving *Water Quality*, *Biodiversity* and *Species Reproduction* contrasts with flexible water allocation seen as a key challenge (*River Flows*, *Collaboration*). *Leadership* is not regarded as a priority challenge, with most scores barely rating 0.5, except for scientists, who may be slightly more critical. The centralist top-down governance framework is viewed by most actor groups as the best way to achieve basin-wide outcomes, although there is a recognition that local water user needs remain problematic (Table 2). This view stands in contrast with the diagnostic scores and the indicator priorities used for enabling pathways.

The Adour-Garonne reveals a similar negative score by irrigators for *Species Reproduction* and *Water Quality*, but also *River Flows* (Fig. 3). This reflects the key challenge of managing *River Flows* for climate change, where the expectation is that natural flows cannot be restored and adaptation strategies for a new ‘normal’ are required. Given the vision for the basin which includes improvement of *Water Quality*, restoration of wetlands and base flow levels (*River Flows*) (Table S2), this points to the lowest of all vision alignment scores of all actor groups (note: *n* = 2). The Adour-Garonne is the basin with the widest divergence of scores, significantly higher than any of the other river basins and indicator SD values being high for all but two indicators (*Leadership* and *Institutions*) (Table 3). The key challenges identified by the various groups suggest that confidence in those two indicators remains high, but deficiencies in *Collaboration* and *Learning* result in a lack of alignment of strategic direction and widely diverging views, some which are more prevalent than others. The European Water Framework Directive (CEC 2000) also imposes an external regulation adding complexity to a governance framework that needs to integrate the big water cycle (river basin scale) with the small water cycle (over which the committee has no influence), according to the RBO subsidiary group (Table 2). These problems are certainly recognised by decision-makers, who score the challenges for all indicators as one of the highest of all actor groups and of all basin profiles.

### Actor contributions to enabling pathways

Designing an enabling pathway, based primarily on what actors consider indicator priorities and complemented by key challenges identified through qualitative questioning is a novel diagnostic approach, which opens new perspectives for co-implementation resulting from indicator interactions and synergies. The diagnosis of current and target condition is used to identify priority indicator targets which may differ for each group depending on their perspective, and how they view indicator interaction influences. By using actor knowledge to assess current and target condition of the basin, actors can be empowered to express a perspective, which sometimes differs from their identified key challenges. Indeed, different forms of knowledge may co-exist and be utilized depending on the context (Delfau 2018). Key challenges may be based on existing narratives, whereas diagnosing key indicators may instead be approached from a problem-solving perspective. Aggregation of responses may also result in differences between key challenges and indicator priorities. Broadening a conceptual understanding became particularly prominent when actors were asked to score influences resulting from indicator interactions. For most participants, influences among socio-institutional and on and among biophysical indicators were easy to articulate. However, scoring the influence of biophysical indicators on socio-institutional indicators was much more challenging, as it required people consider biophysical feedback loops on governance and policy. This is surprising, given that crisis management through policy tools is precisely a response to these biophysical feedback loops.

Diagnosing indicator performance by actors changes the engagement from a purely rational, top-down approach decided by experts and managers to a broader view allowing ‘multiple ways of knowing’ (Barrett et al. 2017; Delfau 2018) and co-learning (Gilfillan et al. 2020). Co-learning changes the dialogue and power dynamics of basin actors in favour of a more egalitarian way in which interaction is no longer uniquely guided by preconceived ideas of users, providers, regulators and managers, but where social capacity aims for transformative sustainability learning (Barrett et al. 2017) and adaptive governance (Folke et al. 2005), requiring longer-term institutional reform (Sharma-Wallace et al. 2018). In this way, co-implementation becomes a concerted effort of all actors, increasing trust, accountability and transparency. It challenges existing governance concepts and compels actors to evaluate system dynamics and feedback loops that include their own position. This process facilitates double and triple loop learning, considered an essential requirement for an adaptive management framework (Pahl-Wostl 2009), which despite its attractiveness has failed to become the dominant framework to date (Connalin et al. 2018).

### Different enabling pathways for basin case studies

The constructed enabling pathways have many implications for these four case study river basins. For the Murray-Darling Basin, the key premise is that priorities need to focus on end objectives of improving *Biodiversity* and *Species Reproduction* to inform targets for the driver indicator *River Flows*, which features prominently in the Basin Plan (2012). This may require broader strategies that consider environmental flows along other natural resource management levers. Institutional reform is an outcome that needs to be driven by end objectives of restoring environmental degradation. It should consolidate a praxis emphasising enhanced *Learning* and *Collaboration*, whilst ensuring effective mechanisms for this are formalised over time (Daniell 2011; Connalin et al. 2018). Accountability for the declining state of the basin has become a pressing issue at the core of evaluating the effectiveness of the Basin Plan and its water recovery for environmental flows (O’Donnell and Garrick 2017; Wentworth Group of Concerned Scientists 2017; Thom et al. 2019; Vertessy et al. 2019), particularly since the balance of societal values has evolved towards sustainable water use (Wei et al. 2017).

In the São Francisco Basin, *Learning*, *Water Quality* and *River Flows* are the main interaction pathways that will bring about improvements to the basin, although only *River Flows* is identified as part of the key challenges (Table 2). Better actor representation and payment for water services will allow many of the basin-wide planning issues to be resolved, and through this bottom-up approach provide for large scale, systemic quantified solutions for water use allocation, Water Pacts, and river basin restoration (Siegmundt-Schultze 2017; Bouckaert et al. 2020). Nevertheless, top-down systemic challenges remain, such as optimizing hydropower to improve biodiversity, a topic not captured in this analysis by absence of this actor group.

The enabling pathway for the Yellow River Basin challenges the hierarchical top-down governance model, by identifying the need for greater *Collaboration* for collective *Learning* to address problems of *River Flows* and *Water Quality.* This pathway may require more inclusive *Leadership* and reform of *Institutions.* Achieving bottom-up horizontal integration where local decision-making feeds into a basin-wide planning framework is part of improved *Collaboration*. This is conveyed in the *Collaboration* to *Learning* to *River Flows* pathway for all actors.

In the Adour-Garonne Basin, decision paralysis, coupled to clearer roles and responsibilities appears to be the constraining issue that challenges the consensus-based governance model (Colon et al. 2018); this was reflected in the answers of many respondents. Interestingly, decision-makers view the challenges for all indicators as much larger than the other groups, suggesting that the process of decision-making itself is the greatest obstacle to improve the indicators. Collectively, actors would require *Leadership* to focus on strategic direction and enhanced *Collaboration*, formalised through reform of *Institutions* in the longer term. Socio-institutional capacity issues dominate multiple pathways, requiring better integration at different levels (local—the small water cycle-, basin-wide -the large water cycle, and international—the EU Water Framework Directive).

In all four river basins, parallels can be drawn between the tension of localised water user needs and top-down planning decisions. In most instances this tension arises from a poorly integrated planning process, where top-down management processes pay insufficient attention to local water requirements and fail to recognise the nested spatial scales requiring subsidiary decision making coupled to input from local actors. To address this issue, it will not be sufficient to simply consult on what actors perceive as shortcomings based on their immediate needs. Instead, actors will need to take on an increasingly proactive role, in which they use river basin diagnosis to look beyond their own agendas and be willing to take on a stronger role in co-implementation as part of a mutual obligation principle, while simultaneously challenging hierarchical decision-making. Indicators of *Learning* and *Collaboration* can be used to exchange information between the four basins on the process of actor basin diagnosis, if this method would become more widely adopted. Existing governance models and its history in each of the basins suggest that *Institutions* and *Leadership* will focus on basin-specific challenges. Nevertheless, relevant lessons can be shared, especially in the context of how external drivers affect a basin system.

### International relevance

Having developed an integrated tool for diagnosing governance and management of river basins offers opportunities for cross-basin comparisons. It could initiate a process of cross-basin learning complementing the OECD principles on water governance in practice (Neto et al. 2018). The most obvious starting point is through connections formed between RBOs, but with the potential to expand it into much larger collaborative networks. In our study, two river basins have similar governance structures of representative actor membership: the São Francisco Basin and the Adour-Garonne Basin. Comparisons of enabling pathways, similarities and differences could provide valuable lessons with regard to actor representation and leadership direction. The other two basins, the Murray-Darling Basin and the Yellow River Basin, have a top-down governance structure, and both identify challenges with compliance issues. Further, comparisons between underrepresented indigenous actors in the Murray-Darling Basin and the São Francisco basin would benefit from the diagnostic approach but would require much larger representative samples of indigenous actors in a separate category. Cross-comparisons of different governance models would also inform how similar challenges are approached, and what could be learnings from legislated representation versus administrative governance accountabilities. This could also reveal some interesting findings on actor representation of the poor in the São Francisco and Yellow River basins in the context of trade-offs made for macroeconomic development. Tangible achievements such as large-scale riverbank revegetation in the Yellow River Basin could also be evaluated through the diagnostic approach, with valuable lessons for other river basins.

In an international context, the diagnostic approach may offer a novel pathway for screening collaboration potential that differs from current conventional narratives of ‘north–south’ international collaboration. This could include comparisons between riparian countries of transboundary rivers, although these are inherently more complex and will need to consider multiple dimensions, including upstream–downstream dependencies, limited power of bilateral and multilateral agreements under international law, interests from donor countries, etc. (Milman et al. 2013; Tilleard and Ford 2016). In this study, the framework was applied at a nationally bounded basin scale, but it can be extended to applications at multiple nested spatial scales, including transboundary rivers.

### Limitations and perspectives

Interviewing basin actors is resource intensive. It provided a wealth of information, yet at the same time limits the number of actors that could be interviewed in each of the basins. Hence results of actor diagnostic scores should be interpreted with caution with regard to representative views based on a small sample of a much larger actor group. The classification of actor groups could also be reviewed, to include indigenous participants and perhaps include a gender-based perspective. Nevertheless, the major contribution of this study is that actor diagnostics provide wider perspectives to discuss actor participation in co-implementation of basin governance and management, and provides comparable indicators across river basins. The suggested approach requires actors to broaden their perspectives on basin management and on their defined roles, be it as decision-maker, practitioner, beneficiary or investigator. Systemic comparisons across river basins of governance performance and its interaction with biophysical condition indicators can assist with a critical analysis of a basin governance framework for achieving sustainable management outcomes. In this study, only five of the ten identified actor groups had data for all four river basins. This excluded some actor groups which may have been important in the debate about implementing a river basin plan, and hence this comparison may have skewed some of the key issues for a basin. For example, in the São Francisco Basin the hydropower supply group was not included in this study (due to absence of comparable groups in other basins), despite a salient debate on hydropower security and its trade-off against improving biodiversity in the management of the basin (O’Hanley et al. 2020).

In a broader sense, the influence of external drivers and context on a river system are included in the conceptual framework and evaluated through qualitative open-ended questions, but not assessed as part of the quantitative score diagnostic. Distinguishing what lies within the realm of influence of an RBO and its actors may need to be clarified, as well as agreeing on a strategy for longer term engagement and repeat diagnostics as part of regular evaluations. The transformation of diagnostics into enabling pathways for engagement would need to become the next step in a social learning process for getting buy-in from participants.

## Conclusions

This research developed an actor diagnostic approach of four social-institutional and four biophysical functional indicators for designing an enabling pathway of river basin governance for sustainable management. The relative importance of functional indicators and their influence on others while progressing towards target perceived by actors was used to provide an interactive pathway for actor groups to engage in co-implementation of their basin plan. Constructing enabling pathways provides a methodology to encapsulate emerging properties of a social dynamic that otherwise may not be considered in the logical planning of a river basin. Using actor diagnostics provide insights into their own positioning in the prevalent governance framework, and an ability to reflect and compare this across basins. The next logical step is the enactment of these pathways to initiate a social learning process as a first step in co-implementation by multiple actor groups of a basin plan. Enactment of these pathways for co-implementation is beyond the scope of this study, but suggested mechanisms are prevalent in the literature, including collaborative governance, co-engineering, scenario praxis), and integrative learning.

## Supplementary Information

Below is the link to the electronic supplementary material.

Supplementary file1 (PDF 1837 kb)
