# Supplementary material for: River basin governance enabling pathways for sustainable management: A comparative study between Australia, Brazil, China and France
Source: Ambio. 2022 Mar 22;51(8):1871–88. doi: 10.1007/s13280-021-01699-4 (PMC9200927; doi:10.1007/s13280-021-01699-4)

Title: Comparing Enabling pathways for sustainable river basin governance and management: case studies from Australia, Brazil, China and France

Authors: Frederick Bouckaert, Yongping Wei, Jamie Pittock, Vitor Vasconcelos and Ray Ison

Table S1 Standardised list of interview questions

Interview questions	Description of categories, dimensions and rating scales
1. Provide your name, title and position role and identify which actor category applies to you:<br>(Note: participant contribution will be anonymised, but this information is important to understand your response)	1. Hydropower/water supply<br>2. Irrigator<br>3. Income livelihood dependent on water<br>4. Community member living in the basin<br>5. General public<br>6. NGO<br>7. Scientist<br>8. Decision-maker<br>9. RBO staff<br>10. SBO subsidiary (other government involved in water governance)
2. Describe your vision for the [*relevant*] basin.<br>In preparation for the next question, the vision of the basin, as defined by the RBO or relevant policy, is read out to participant after the answer is received.	Key elements to consider:<br>What to achieve? Why? For whom? How? Timeframe? By whom?
3. How strong do you think you vision aligns with that of your RBO/River basin? (rate). What are the major differences? (Qualitative response)	1. Very weak<br>2. Weak<br>3. Moderate<br>4. Strong<br>5. Very strong
4. How strong do you think your personal influence is (within the organisation you work for) in contributing to river basin management? (rate)<br>5. How strong do you think your organisation influence is in contributing to river basin management? (rate)	1. Very weak<br>2. Weak<br>3. Moderate<br>4. Strong<br>5. Very strong

6.	What are the opportunities and limitations of the RBO in managing contemporary threats to river basins? Consider three aspects:	Contemporary threats:<br>RBO opportunities to manage:<br>RBO limitations in managing:
7.	How do river basin governance arrangements evolve over time to reflect shifting and competing priorities with respect to delivering ecosystem services?	A series of policy initiatives are provided, relevant to each river basin case study; these often include the start of a basin plan. Comments are invited.
8.	How can RBO governance arrangements and ecosystem management objectives be made 'fit for purpose' across different social/cultural, environmental, economic and political circumstances?	Social/cultural:<br>Environmental:<br>Economic:<br>Political:<br>Across all dimensions:
9.	How can RBO governance arrangements and ecosystem management objectives be made 'fit for purpose' across different spatial scales (including tributaries)	Upper, middle and lower are spatial categories, but they may be tailored to the case study (e.g. in the Murray Darling, upper, middle and lower is listed for both the Darling and for the Murray).
10.	Score the framework indicators for current condition for each of the eight indicators: Leadership, Collaboration, Institutions, Learning, Water quality, River Flows, Biodiversity, Species Reproduction.	1. Very Poor<br>2. Poor<br>3. Moderate<br>4. Good<br>5. Very good
11.	Score the framework indicators for target condition. Target condition is set at completion of the basin plan, or in absence of this, a ten-year time frame.	A descriptive rubric is provided to assist with the scoring for both current and target condition (Table S4).

12. If one indicator improves towards success target, how does this influence the other indicators?	A matrix is provided, and the participant is guided through the scoring of influence of indicator A on B etc. The rating scale is:<br>-2 Counteracting<br>-1 Constraining<br>0 Neutral/No influence<br>1 Enabling<br>2 Reinforcing

13. Describe your key findings/reflections and conclusions, main thoughts.

14. How useful do you think this framework is in assisting with river basin governance? Why or why not?

At this point, the participant is also asked if they can suggest another relevant potential participant for interviewing (Snowball sampling method, Cohen & Arieli, 2011). They are then thanked for their time and this is the end of the interview.

Table S2 Vision and mission statements for each of the four basin case studies

São Francisco Basin	Murray-Darling Basin	Yellow River Basin	Adour-Garonne Basin
Vision: To promote an integrated approach to the planning and management of the São Francisco River Basin.	Vision: To achieve a health working basin through the integrated management of water resources for the long-term benefit of the Australian community*	Vision: Plan of ensuring the integrity of river ecosystem functions in the Yellow River Basin (2010).	Vision: the 10th programme has defined for 6 years (2013-2018) the interventions of the Agency for:<br>• Improvement of water quality for human consumption, especially with regard to non-point source pollution<br>• Restoration of the functions of wetlands<br>• Maintaining adequate water levels for rivers and wetlands, in particular base flow levels in the context of climate change

Mission:<br>• Decentralising the power of decision<br>• Integrate public and private actions<br>• Promote participation of all sectors of society	Mission: We lead the planning and management of Basin water resources in collaboration with partner governments and the community	Mission: to implement the Strict Water Resource Management Policy (SWRM) and the priorities of water (3 red lines):<br>• Optimize the allocation and water efficiency of resources<br>• Promote water saving<br>• Improve ecosystems and control water pollution	Mission of the SDAGE management plan (2016-2021):<br>• To create favourable governance conditions<br>• To reduce pollution and improve quantitative management<br>• To preserve and restore aquatic environments.

Table S3 Policy reform initiatives for each of the four river basins

São Francisco Basin	Murray-Darling Basin	Yellow River Basin	Adour-Garonne Basin
• Predevelopment phase (entangled institutions); before 1997 • Water Law, IWRM, River Basin Committees (1997)<br>• Transposition (water transfer) and revitalisation (river restoration) (2007)<br>• Plano de recursos hidricos (Basin Plan v2) (2016)<br>• End target year for Plano de recursos hidricos (2025)	• Governance arrangement before Basin Plan (prior to 2012)<br>• Governance at start of the Basin Plan (2012)<br>• Governance at full implementation of the Basin Plan (2024)	• Water Law (1988)<br>• Administrative Measures of Yellow River Water Diversion (1988)<br>• Ministry of Water Resources: to ensure the Yellow River (physically) reaches the sea (Revised water Law, 2002)<br>• Maintaining the health of the Yellow River (2004)<br>• Integrated Plan of the Yellow River Basin (2010)<br>• National Water Policy Strict Water Resource Management (2011):<br>• Strengthen water resources development and utilisation and control<br>• Promote water use efficiency<br>• Control of water pollution	• La loi sur l'eau (1964): regional development<br>• Contract de rivière (1984): local development<br>• La nouvelle loi sur l'eau (1992): sustainable development (SDAGE and SAGE management plans)<br>• Directive cadre Européenne (2000): territorial development<br>• La loi sur l'eau et les milieu aquatiques: application of European framework<br>• Plan adaptation au changement climatique (2018): climate change plan

Table S4 Descriptive rubric for diagnostic scoring of current and target capacity

Indicators	***Scoring level***				
	**Very poor**	**Poor**	**Moderate**	**Good**	**Very good**
	**1**	**2**	**3**	**4**	**5**
**Leadership (social driving function)** Authority - Regulatory power – Legal mechanisms – Economic incentives	Leadership functions are fragmented or non-existent, requiring major long-term effort to establish working lines of authority, regulatory powers, legal mechanisms, economic incentives	Some aspects/components of leadership are functioning, but there are major gaps or constraints that impede lines of authority, regulatory powers, legal mechanisms and/or economic incentives.	Leadership functions are existing, but some important shortcomings have been identified around lines of authority, regulatory powers, legal mechanisms and/or economic incentives that need to be addressed	Leadership functions are strong. Some issues around lines of authority, regulatory powers, legal mechanisms and economic incentives are identified that could be improved	Leadership functions are very strong. There are clear lines of authority; regulatory powers, legal mechanisms and economic incentives are well aligned for driving work forward.
**Collaboration (social connecting function)** Strength – Formalisation – Clarity of roles – Transparency	Collaborations functions are fragmented or non-existent, requiring major long-term effort to establish: stronger connections and transparent, trusting relationships; formal lines of communication; and clarity of actor responsibilities	Some aspects/components of collaborations are functioning, but there are major gaps in: stronger connections and transparent, trusting relationships; formal lines of communication; and clarity of actor responsibilities. Connections are based on personal relationships with weak resilience.	Collaboration functions cover most aspects of the basin management; however, there are a number of unresolved issues and there is lack of clarity on actor expectations and whether these are being met.	Collaboration functions are generally strong. There are good connections and transparent, working relationships; formal lines of communication; and clarity of actor responsibilities. Conflict resolution needs strengthening.	Collaboration functions are very strong. There are strong connections and transparent, trusting relationships; formal lines of communication; and clarity of actor responsibilities. Conflict resolution processes exist and are functioning well.
**Institutions (social assemblage function)** Modularity – Self-organising capacity – Accountability – Representation	Institutions are fragmented or non-existent, requiring major long-term effort to establish working governance. No actor analysis exists and representation is inadequate.	Institutions are incomplete, requiring long-term effort to establish working governance. Actor analysis has been completed but many groups still need to be engaged to establish lines of communication.	Institutions are rigid and modularity, self – organising capacity, accountability and representation are rudimentary. Capacity for flexibility and change is limited, and resilience is moderate to low.	Institutions are coherent, with some self-organising capacity related to roles and responsibilities; limited modularity restricts flexibility in time of crisis, but accountability is agreed and shortcomings are understood by all.	Institutions are robust, with adequate self-organising capacity related to roles and responsibilities; modularity allows for flexibility in time of crisis, but accountability is agreed and well understood by all.

**Learning (social renewal function)** Adaptive management – Triple loop learning – Generate and share data & information – Evaluation	Learning is fragmented and hampered by conflicting views and priorities, poor data collection or systemic evaluation. Lack of data and evidence result in distrust.	Learning is fragmented and haphazard; systematic data collection is not fully integrated and comprehensive evidence may be lacking. This may result in contesting narratives based on selective information.	Learning is limited to some key actors, and sharing of information is restricted to formal reporting and evaluation. Potential for adaptive learning and triple loop learning not fully reached.	Learning is characterized by some adaptive management at selected scales, robust data collection and sharing to relevant parties, regular evaluations including triple loop learning.	Learning is characterized by well-established processes for adaptive management at multiple scales, robust data collection and sharing to relevant parties, regular evaluations including triple loop learning.
**Water Quality (biophysical driving function)** Erosion and deposition – Nutrient cycling – Carbon cycling – Water quality	Water quality is seriously impeded. River basin condition is severely degraded and capacity for significant performance improvement is unlikely. Severe impacts are common (e.g. saltwater intrusion, algal blooms, hypoxic events)	Water quality is impeded but less severe and/or frequent impacts are manifesting (e.g. algal blooms, hypoxic events). Performance improvement poses significant challenges.	Water quality is functioning in some cases. Numerous aspects of the system may create barriers to one or more cycling systems (soil, nutrients, carbon, water) causing sporadic impacts (e.g. algal blooms, hypoxic events). Performance improvement requires consistent and on-going effort.	Water quality is functioning in most cases. Some aspects of the system may create barriers to one or more cycling systems (soil, nutrients, carbon, water). Performance improvement may be targeted for positive impact.	Water quality is well-functioning. There are almost no barriers to natural cycling processes. Few performance improvements are required.
**River Flows (biophysical connecting function)** Hydrological connectivity – Provisioning of habitat – Water diversions – Flow regime change	River flows are seriously impeded. Flow regimes are changed irreversibly for the largest part of the river system. There is strong evidence that poor management compounds climate variability.	River flows are impeded. Most flow regimes are compromised, with almost no capacity for restoration in critical parts of the basin.	River flow regimes are compromised , and many parts of the basin display flow changes with limited capacity to manage or restore.	River flows are functioning in a diminished capacity. Some locations have permanent flow changes that are considered irreversible, due to need for human consumption. Generally, most flow regimes have been maintained.	River flows are well-functioning, with most habitats being restored, and plant and animal requirements being met. There are almost no barriers to biological distributions. Few performance improvements are required.
**Biodiversity (biophysical assemblage function)**	Biodiversity is severely impoverished, with many species gone	Biodiversity is impoverished, with some key species gone extinct, and pervasive invasions	Biodiversity is in moderate condition, with some biological	Biodiversity is in good condition, with some biological hotspots and ecological	Biodiversity is in optimal condition, with most biological hotspots and ecological

**Biodiversity** – ES accounting – Rare species and ecosystems – Exotic invasions	extinct, and pervasive invasions of exotic species. Insufficient buffer zones exist, with large parts of permanently changed habitat.	of exotic species. Buffer zones are often infested with exotic species, and moderate parts of permanently changed habitat.	hotspots and ecological assets in moderate condition and some threatened species. Evidence shows that for some, their status is declining and some pervasive exotic species out compete native plants and animals.	assets in good condition and few threatened species. Evidence shows that their status is maintained, across most significant ecosystem components (vegetation, fish, macroinvertebrates, water birds, and other groups)	assets in good condition and very few threatened species. Evidence shows that their status is maintained, across all significant ecosystem components (vegetation, fish, macroinvertebrates, water birds, and other groups). Almost no presence of invasive species.
**Species Reproduction (biophysical renewal function)** Pathways and adequate flows – Hydraulic regime – Dispersal mechanisms – Invasive species	Species reproduction is severely impaired, evidenced by uneven age cohort distributions in many significant locations. Historical evidence suggests this has led to extinctions in the past. Landscape is severely fragmented with no corridors for dispersal remaining.	Species reproduction is impaired, evidenced by uneven age cohort distributions in many significant locations. Landscape is fragmented with few corridors for dispersal remaining. Long term sustainability prospects are looking grim.	Species reproduction is functioning but some key species show recruitment impairment. Landscape fragmentation is fairly significant, and there is a concern for long term sustainability of some key species.	Species reproduction is functioning for most key species, evidenced by different age cohort information across most key species for which data have been collected. Corridors exist for dispersal, but some fragmentation of the landscape exists.	Species reproduction is well functioning, evidenced by different age cohort information across most key species for which data have been collected. Significant corridors exist for dispersal, and species are healthy and showing no signs of stress

Fig. S1 Current and target scores by basin and actor group, used to derive (T-C) scoring profiles (Fig. 3)

River basin	Stakeholders	Leadership	Collaboration	Institutions	Learning	Water Quality	River Flows	Biodiversity	Species Reproduction
Murray-Darling Basin current condition	Decision-maker	3.90	2.67	3.40	3.60	2.33	3.00	2.17	2.67
	Irrigator	3.00	3.00	2.67	2.83	2.33	2.50	1.67	2.00
	NGO	1.60	2.20	2.40	1.90	1.60	1.70	1.70	2.00
	RBO subsidiary	2.13	2.75	2.88	2.50	2.75	2.75	2.25	2.38
	Scientist	2.63	2.44	3.13	2.50	2.19	1.94	2.13	2.25
	All	2.65	2.61	2.89	2.67	2.24	2.38	1.98	2.26
Murray-Darling Basin target condition	Decision-maker	4.00	3.67	4.00	4.33	3.67	4.07	3.67	3.67
	Irrigator	4.00	4.00	4.17	4.17	3.67	4.00	3.67	3.67
	NGO	4.10	4.50	4.50	4.30	3.90	4.10	3.90	3.90
	RBO subsidiary	4.25	4.25	4.25	4.50	4.00	4.00	3.75	3.63
	Scientist	3.94	3.56	4.13	4.06	3.31	3.31	3.13	3.56
	All	4.06	4.00	4.21	4.27	3.71	3.90	3.62	3.68
São Francisco Basin current condition	Decision-maker	3.33	3.00	3.33	3.33	2.33	2.83	2.67	2.33
	Irrigator	3.50	2.88	3.00	3.25	1.75	3.00	2.38	2.50
	NGO	3.75	3.88	3.38	3.75	1.88	1.75	2.00	1.75
	RBO subsidiary	3.00	2.88	2.25	2.63	2.00	2.25	2.25	2.00
	Scientist	3.67	2.78	2.89	3.11	1.67	2.39	2.17	1.94
	All	3.45	3.08	2.97	3.21	1.93	2.44	2.29	2.11
São Francisco Basin target condition	Decision-maker	4.33	4.00	3.67	4.50	3.33	3.50	3.00	3.00
	Irrigator	4.50	3.88	4.13	4.00	3.50	4.25	3.63	3.75
	NGO	3.50	3.88	3.88	4.25	2.75	2.75	2.00	3.00
	RBO subsidiary	4.25	3.75	3.75	4.00	3.25	3.38	3.25	3.50
	Scientist	4.56	4.11	4.50	4.33	3.11	3.72	2.94	2.61
	All	4.23	3.92	3.98	4.22	3.19	3.52	2.96	3.17
Yellow River Basin current condition	Decision-maker	4.6	4.0	4.3	3.9	3.3	3.6	3.5	3.8
	Irrigator	4.0	3.0	3.0	3.5	3.5	4.0	4.0	5.0
	NGO	5.0	2.0	2.5	3.5	3.0	3.0	3.0	3.0
	RBO subsidiary	4.3	3.3	3.8	3.8	3.3	3.2	3.6	3.5
	Scientist	3.8	3.0	3.3	3.3	3.5	3.3	3.0	2.5
	All	4.3	3.1	3.4	3.6	3.3	3.4	3.4	3.6
Yellow River Basin target condition	Decision-maker	4.8	4.8	4.8	4.4	4.3	4.3	4.3	4.5
	Irrigator	4.5	4.5	3.5	4.5	3.5	4.5	4.0	5.0
	NGO	5.0	3.5	3.5	5.0	4.0	4.0	4.0	4.0
	RBO subsidiary	4.7	4.5	4.5	4.2	3.8	4.0	4.4	4.3
	Scientist	4.8	4.0	4.0	4.8	4.0	4.3	3.8	3.8
	All	4.7	4.3	4.1	4.6	3.9	4.2	4.1	4.3
Adour-Garonne Basin current condition	Decision-maker	3.50	2.50	3.00	2.75	2.75	2.63	2.25	2.25
	Irrigator	3.50	2.00	3.25	3.25	3.75	4.25	3.50	4.25
	NGO	2.75	2.88	2.75	3.50	2.38	2.75	3.25	3.13
	RBO subsidiary	3.50	3.10	3.40	2.90	2.50	3.20	3.30	2.90
	Scientist	3.50	4.50	3.00	3.50	3.25	3.00	4.25	3.25
	All	3.35	3.00	3.08	3.18	2.93	3.17	3.31	3.16
Adour-Garonne Basin target condition	Decision-maker	5.00	4.50	4.50	4.50	4.75	4.25	4.25	4.25
	Irrigator	4.75	3.25	4.75	4.25	3.00	3.00	3.75	3.75
	NGO	4.50	4.25	4.00	4.50	3.88	3.75	3.00	3.63
	RBO subsidiary	4.40	4.00	4.10	4.60	3.70	3.40	3.60	3.40
	Scientist	4.50	5.00	4.00	3.50	4.50	4.00	4.50	4.00
	All	4.63	4.20	4.27	4.27	3.97	3.68	3.82	3.81

Fig. S2 Summary table of indicator rank order values, highest interactive influence value and highest cumulative interactive influence value, used in enabling pathways design.

Data analysis Step 1: Rankorder Largest (T_{score} - C_{score})

River Basin	Stakeholder groups	Leadership	Collaboration	Institutions	Learning	Water Quality	River Flows	Biodiversity	Species Reproduction
Murray-Darling Basin	Scientists	3			1		2		3
Murray-Darling Basin	Decision-makers					2	3	1	
Murray-Darling Basin	Irrigation						3	1	2
Murray-Darling Basin	NGO	1	3				2		
Murray-Darling Basin	RBO Subsidiary	1			2			3	
Murray-Darling Basin	All				2	3		1	
São Francisco River Basin	Scientists		3	1		2	3		
São Francisco River Basin	Decision-makers	2	2		1	2	3		3
São Francisco River Basin	Irrigation			3		1	2	2	2
São Francisco River Basin	NGO	<0				3	2		1
São Francisco River Basin	RBO Subsidiary	3		1	2	3			1
São Francisco River Basin	All					1	2		3
Yellow River Basin	Scientists	3		3	1		3		2
Yellow River Basin	Decision-makers		2			1	3	2	2
Yellow River Basin	Irrigation	3	1	3	2		3		
Yellow River Basin	NGO		1	2	1	2	2	2	2
Yellow River Basin	RBO Subsidiary		1	3			2	2	2
Yellow River Basin	All		1		2		3		
Adour-Garonne Basin	Scientists	2		2		1	2		3
Adour-Garonne Basin	Decision-makers		1		2	1	3	1	1
Adour-Garonne Basin	Irrigation	2	2	1	3	<0	<0		<0
Adour-Garonne Basin	NGO	1	2	3				<0	
Adour-Garonne Basin	RBO Subsidiary	3	3		1	2			
Adour-Garonne Basin	All	1	2	3					

Data analysis Step 2: summed largest influence on other indicator

River Basin	Stakeholder groups	Leadership	Collaboration	Institutions	Learning	Water Quality	River Flows	Biodiversity	Species Reproduction
Murray-Darling Basin	Scientists		1.8						
Murray-Darling Basin	Decision-makers				2.0				
Murray-Darling Basin	Irrigation				2.0			-0.3	
Murray-Darling Basin	NGO			1.4		1.4	1.4	1.4	1.4
Murray-Darling Basin	RBO Subsidiary	1.5	1.5				1.5		
Murray-Darling Basin	All		1.6						
São Francisco River Basin	Scientists		1.7		1.7	1.7			
São Francisco River Basin	Decision-makers	2.0	2.0	2.0		2.0	-0.3	-0.3	-0.3
São Francisco River Basin	Irrigation				2.0			2.0	
São Francisco River Basin	NGO		2.0			2.0			2.0
São Francisco River Basin	RBO Subsidiary							1.9	
São Francisco River Basin	All				1.8				
Yellow River Basin	Scientists						1.8	1.8	1.8
Yellow River Basin	Decision-makers		2.0						
Yellow River Basin	Irrigation		2.0		2.0				
Yellow River Basin	NGO		1.5	1.5	1.5				
Yellow River Basin	RBO Subsidiary							1.5	1.5
Yellow River Basin	All		1.3	1.3	1.3				
Adour-Garonne Basin	Scientists						2.0	2.0	2.0
Adour-Garonne Basin	Decision-makers				2.0				
Adour-Garonne Basin	Irrigation		2.0	2.0			2.0		
Adour-Garonne Basin	NGO						2.0		
Adour-Garonne Basin	RBO Subsidiary						1.8		
Adour-Garonne Basin	All							2.0	

Data analysis Step 3: Cumulative strongest influence (three largest values)

River Basin	Stakeholder groups	Leadership	Collaboration	Institutions	Learning	Water Quality	River Flows	Biodiversity	Species Reproduction
Murray-Darling Basin	Scientists		7.9						
Murray-Darling Basin	Decision-makers				**9.3**				
Murray-Darling Basin	Irrigation		**9.7**						
Murray-Darling Basin	NGO				8.0				
Murray-Darling Basin	RBO Subsidiary	**9.0**							
Murray-Darling Basin	All				8.5				
São Francisco River Basin	Scientists				9.4				
São Francisco River Basin	Decision-makers			**11.7**					
São Francisco River Basin	Irrigation				9.7				
São Francisco River Basin	NGO								**10.8**
São Francisco River Basin	RBO Subsidiary		9.1						
São Francisco River Basin	All				**9.8**				
Yellow River Basin	Scientists			7.8					
Yellow River Basin	Decision-makers	**9.0**							
Yellow River Basin	Irrigation	**10.0**							
Yellow River Basin	NGO				8.0				
Yellow River Basin	RBO Subsidiary				**8.2**				
Yellow River Basin	All	7.3							
Adour-Garonne Basin	Scientists		6.5						
Adour-Garonne Basin	Decision-makers				**12.3**				
Adour-Garonne Basin	Irrigation						**10.5**		
Adour-Garonne Basin	NGO				**12.0**				
Adour-Garonne Basin	RBO Subsidiary				6.6				
Adour-Garonne Basin	All					8.4		8.4	

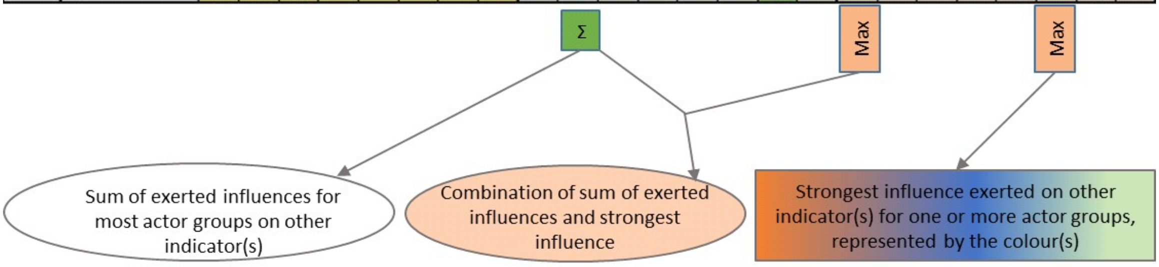

Supplement: Supplementary file 1 — Supplementary file1 (PDF 1837 kb) [file 13280_2021_1699_MOESM1_ESM.pdf]
